# Silibinin inhibits the production of pro-inflammatory cytokines through inhibition of NF-κB signaling pathway in HMC-1 human mast cells

**DOI:** 10.1007/s00011-013-0640-1

**Published:** 2013-09-18

**Authors:** Beom-Rak Kim, Hye-Sook Seo, Jin-Mo Ku, Gyung-Jun Kim, Chan Yong Jeon, Jong Hyeong Park, Bo-Hyoung Jang, Sun-Ju Park, Yong-Cheol Shin, Seong-Gyu Ko

**Affiliations:** 1 Department of Oriental Medicine, Gachon University, 1342 Seongnamdaero, Sujeong Gu, Seongnam, Gyeonggi Do 461-701 Republic of Korea; 2 Laboratory of Clinical Biology and Pharmacogenomics and Center for Clinical Research and Genomics, Institute of Oriental Medicine, Kyung Hee University, 26 Kyungheedae-ro, Dongdaemun-gu, Seoul, 130-701 Republic of Korea

**Keywords:** Silibinin, *Cirsium japonicum*, Allergic inflammation, Human mast cells, NF-κB, Pro-inflammatory cytokine

## Abstract

**Background:**

Silibinin is the major active molecule of silymarin, the mixture of flavonolignans extracted from *Cirsium japonicum*. It has been used for the treatment of hepatitis and inflammation-related diseases. In the present study, the effects of silibinin on allergic inflammation and its signaling were investigated in the induced human mast cells.

**Methods:**

Cell growth inhibition induced by silibinin was measured by MTS assay. Histamine release was measured by enzyme immunoassay. The tumor necrosis factor-α (TNF-α), interleukin-6 (IL-6), and interleukin-8 (IL-8) secreted protein levels and mRNA levels were measured by the ELISA assay and RT-PCR, respectively. The NF-κB promoter activity was examined by a luciferase assay.

**Results:**

Silibinin suppressed the growth of HMC-1 cells and also reduced the production and mRNA expression of pro-inflammatory cytokines such as TNF-α, IL-6, and IL-8. Moreover, silibinin inhibited the nuclear translocation of nuclear factor (NF)-κB through inhibition of the phosphorylation of IκBα and suppressed NF-κB transcriptional activity in stimulated HMC-1 cells.

**Conclusions:**

Taken together, these results indicate that silibinin inhibits the production of pro-inflammatory cytokines through inhibition of NF-κB signaling pathway in HMC-1 human mast cells, suggesting that silibinin could be used for the treatment of mast cell-derived allergic inflammatory diseases.

## Introduction

Inflammation is part of the complex biological response of vascular tissues to harmful stimuli, such as pathogens, damaged cells, or irritants [1]. This is related to cytokines and pro-inflammatory mediators secreted from macrophage. An allergic reaction is the result of an inappropriate immune response triggering inflammation [2]. A common example is hay fever, which is caused by a hypersensitive response by skin mast cells to allergens [3]. In allergic inflammation, humans produce immunoglobulin E (IgE) against allergen infiltration resulting in activation of mast cells which release histamine, tumor necrosis factor-α (TNF-α), interleukin-6 (IL-6), interleukin-8 (IL-8), and NF-κB [4]. Histamine is produced by basophils and by mast cells found in nearby connective tissues [5]. Histamine binds to H1 receptors in the target cells to contract gut and bronchus smooth muscle and to increase venuli permeability and rheum [6]. Histamine increases the permeability of the capillaries to white blood cells and some proteins to allow them to engage pathogens in the infected tissues [6]. TNF-α is secreted from the allergic mechanism of mast cells, macrophage, and T cells, causes the expression of adhesion factor to vascular endothelial cells, and accumulates white blood cells, resulting in inflammation response [7, 8]. IL-6 causes chronic inflammatory response, activating T cells and producing IgE [9]. Il-8 acts as a chemotactic factor for neutrophil, eosinophil, and T-lymphocyte, activating inflammatory response [10]. NF-κB functions as a transcription factor binding to the NF-κB response element located at the promoter of target genes regulating TNF-α, IL-6, and IL-8 [11, 12]. The secretion of histamine, TNF-α, IL-6, IL-8, and NF-κB is important in inflammatory response and their appropriate regulation could help in treating inflammatory disease.

*Cirsium japonicum* (CJ) is a wild perennial herb native to Korea, Japan, and China. It has been used as an anti-hemorrhagic, anti-hypertensive, and anti-hepatitis agent in traditional Chinese medicine [13, 14]. Recent studies have found that the water extracts of CJ induce the activation of estrogen receptors and have estrogenic effects [13, 15]. CJ also contains a vasorelaxant principle, mediating histamine H1-receptor activation [13, 16]. CJ has been prescribed in the treatment of tumors, such as liver cancer, uterine cancer, and leukemia [17, 18], and used as a hemostatic agent in herbal preparations to prevent epistaxis and metrorrhagia and to improve blood circulation [17]. Of note, silibinin, the major active molecule of silymarin, the mixture of flavonolignans extracted from CJ, is a very strong antioxidant compound capable of scavenging both free radicals and reactive oxygen species. Silibinin protects mice from T cell-dependent liver injury [19]. A recent report in rodents suggested that silibinin may be useful in the chemoprevention of malignancies in the skin, bladder, liver, cervix, and colon [20, 21]. Silibinin induced autophagic and apoptotic cell death in HT1080 cells through a reactive oxygen species pathway [22].

In the present study, we investigated whether CJ and silibinin has a therapeutic effect on allergic inflammatory disease. For that purpose, we measured the levels of histamine, TNF-α, IL-6, and IL-8 in human mast cells, HMC-1 activated by PMA plus A23187 under treatment with CJ and silibinin. We found that silibinin decreased histamine release and reduced the production and mRNA expression of TNF-α, IL-6, and IL-8, while CJ did not show such effects. We also found that silibinin blocked nuclear translocation of NF-κB inhibiting the phosphorylation of IκBα and suppressed NF-κB transcriptional activity in stimulated HMC-1 cells. This suggests that silibinin inhibits the allergic inflammatory response through inhibition of the NF-κB signaling pathway in HMC-1 human mast cells.

## Materials and methods

### Preparation of CJ ethanol extract

CJ used in this study was purchased from Omniherb (Yeongcheon, Republic of Korea). A ground powder of a mass of 100 g was extracted twice with 80 % (V/V) ethanol by using an ultra-sonicator (Branson Ultrasonics, Danbury, CT, USA) for 30 min at room temperature. The resulting extract was then filtered through a 0.22-μm filter and concentrated to approximately 100 ml under reducing pressure. The ethanol extract was evaporated at 40 °C (Evaporator, Eyela, Japan) and then freeze-dried for 72 h (Freezedryer, Matsushita, Japan). The powder from the extract was dissolved in DMSO and stored in aliquots at −80 °C until further analysis.

### Reagents

Iscove’s modified Dulbecco’s medium (IMDM), Dulbecco’s modified Eagle’s medium (DMEM), fetal bovine serum (FBS), antibiotic–antimycotic, and phosphate-buffered saline (PBS) were purchased from Gibco-BRL (Rockville, MD, USA). Phorbol 12-myristate 13-acetate (PMA), A23187, lipopolysaccharide (LPS), and silibinin were obtained from Sigma-Aldrich (St. Louis, MO, USA). MTS assay kit and histamine assay kit were purchased from Promega (Madison, WI, USA) and Oxford Biomedical Research (Oxford, MI, USA), respectively. Luciferase assay system was from Promega (Madison, WI, USA). EZ-western detection kit was obtained from Daeillab (Daeillab Service Co., Seoul, Korea).

### Antibodies

Anti-human TNF-α antibody, biotinylated anti-human TNF-α antibody, and human TNF-α recombinant protein were obtained from R&D Systems (Minneapolis, MN, USA). Anti-human IL-6/IL-8 antibody, anti-mouse IL-6 antibody, biotinylated anti-human IL-6/IL-8 antibody, biotinylated anti-mouse IL-6 antibody, and human IL-6/IL-8 recombinant protein, mouse IL-6 recombinant protein were from BD Biosciences (San Diego, CA, USA). Antibodies against NF-κB, IκBα, and p-IκBα were purchased from Santa Cruz Biotechnology, Inc (Santa Cruz, CA, USA).

### Cell culture

Human mast cells, HMC-1 were maintained as monolayer cultures in IMDM that was supplemented with 10 % FBS, 1 % antibiotic–antimycotic at 37 °C in a humidified incubator under 5 % CO_2_ gas. RAW264.7 mouse macrophage cells were obtained from the Korea Cell Line Bank (Seoul, Korea). Cells were cultured in DMEM supplemented with 10 % FBS, 1 % antibiotic–antimycotic at 37 °C in a 5 % CO_2_ humidified incubator.

### MTS assay

HMC-1 cells or RAW264.7 cells were seeded at a density of 1 × 10^6^ cells/well in 24-well plates, pretreated with various concentrations of CJ (0.05–0.4 mg/ml), and/or silibinin (0.05–0.4 mM) for 1 h and then incubated for 24 h in the absence or presence of PMA (25 nM) plus A23187 (1 μM) or LPS (1 μg/ml). After 24 h of incubation, MTS reagents were added to the culture medium before detection of absorbance at 490 nm. Since the absorbance correlates to the viability of cells, cell viability (%) was calculated using the following formula: cell viability (%) = (absorbance of cells treated with CJ or silibinin − absorbance of blank well)/(absorbance of control cells − absorbance of blank well) × 100.

### Histamine release measurement

HMC-1 cells were preincubated with silibinin for 1 h and then incubated with PMA and A23187 for 6 h. The culture medium was submitted to Enzyme immunoassay kit (Oxford Biomedical Research, Oxford, MI, USA) to measure histamine release.

### The measurement of pro-inflammatory cytokines (TNF-α, IL-6, IL-8)—ELISA (enzyme-linked immunosorbent assay)

The HMC-1 cells or RAW264.7 cells (1 × 10^6^ cells) were incubated with various concentrations of CJ (0.05–0.2 mg/ml) or silibinin (0.05–0.2 mM) for 1 h and then treated with PMA plus A23187 or LPS for 24 h. To measure pro-inflammatory cytokines, 96-well plates were coated with anti-human TNF-α, IL-6, and IL-8 monoclonal antibody and anti-mouse IL-6 monoclonal antibody in 0.1 M sodium carbonate buffer (pH 9.5) and then incubated overnight at 4 °C. After washing the wells, the plates were blocked with 10 % FBS in PBS and then incubated at room temperature for 1 h. After additional washing, samples (culture supernatants) were incubated for 2 h at 37 °C and washed with PBS containing 0.05 % Tween 20 (PBST), and then incubated with each of 0.2 μg/ml biotinylated anti-human TNF-α, IL-6, and IL-8 and anti-mouse IL-6 at room temperature for 1 h. Incubation with streptavidin-horseradish peroxidase and subsequent treatment with tetramethylbenzidine and hydrogen peroxide substrate in the dark were done for each 30 min together with washing, then stopped using 2NH_2_SO_4_. Color development was measured using a microplate reader at 450 nm. The inhibition percentage of cytokine production was calculated using the following equation: % inhibition = (*A* − *B*) × 100/A, where *A* and *B* were the cytokine production without and with CJ or silibinin, respectively.

### RNA extraction and reverse transcription-polymerase chain reaction (RT-PCR)

Total cellular RNA was isolated using an easy-BLUETM RNA extraction kit (iNtRON Biotech, Republic of Korea) according to the manufacturer’s instructions. Total RNA (2 μg) was synthesized to cDNA using M-MLV reverse transcriptase (Invitrogen, Grand Island, NY) according to the manufacturer’s instructions. PCR was conducted out in a 20-μl reaction mixture consisting of cDNA template, 10 pmol of each gene-specific primer, 10× *Taq* buffer, 2.5 mM dNTP mixture, and 1 unit of *Taq* DNA polymerase (Takara Korea, Seoul, Korea). PCR was performed using the following primers for TNF-α (5′-TGAGCACTGAAAGCATGATCC-3′, 5′-ATCACTCCAAAGTGCAGCAG-3′), IL-6 (5′-AACCTTTCCAAAGATGGCTGAA-3′, 5′-CAGGAACTGGATCAGGACTTT-3′), IL-8 (5′-TCAGTGCATAAAGACATACTCC-3′, 5′-TGGCATCTTCACTGATTCTTG-3′), and GAPDH (5′-CGTCTTCACCACCATGGAGA-3′, 5′-CGGCCATCACGCCACAGTTT-3′). The sequencing involved thermal cycling at 95 °C for 1 min (denaturation), 50 °C for 1 min (annealing), and 72 °C for 1 min (extension). The products were checked by agarose electrophoresis and analyzed using the ChemiDoc imaging system.

### Preparation of cytosolic and nuclear protein

Cells were incubated in buffer A (10 mM HEPES, pH 7.9, 10 mM KCl, 1.5 mM MgCl_2_, 0.5 mM dithiothreitol (DTT), and 0.2 mM phenyl-methylsulfonyl fluoride (PMSF). The cells were stranded on ice for 5 min and then centrifuged at 4,000 × *g* for 5 min. The pellet was then lysed with buffer B (10 mM HEPES, pH 7.9, 10 mM KCl, 1.5 mM MgCl_2_, 0.1 % NP-40, 0.5 mM DTT, and 0.2 mM PMSF) and centrifuged at 4,000 × *g* for 5 min at 4 °C. The cytoplasmic proteins were extracted from the supernatant. The pellet was resuspended in Buffer C (20 mM HEPES, pH 7.9, 420 mM NaCl, 1.5 mM MgCl_2_, 25 % glycerol, 0.2 mM EDTA, 0.5 mM DTT, and 0.2 mM PMSF) and incubated on ice for 30 min, and then centrifuged at 4,000 × *g* for 10 min at 4 °C. Nuclear proteins were obtained from the supernatant.

### Western-blot analysis

An equal amount of protein in total cell extracts was separated by SDS-PAGE. After electrophoresis, the proteins were transferred to nitrocellulose membrane (Schleicher & Schuell Bioscience, Dassel, Germany). The membrane was blocked, incubated overnight at 4 °C with primary antibodies (anti-NF-κB, anti-p-IκBα, and anti-IκBα), washed with PBS-0.1 % Tween-20 (PBS-T), and incubated with appropriate HRP-conjugated secondary antibodies at room temperature for 1 h. Immunoreactive protein was developed using an EZ-western detection kit.

### NF-κB reporter activity assay

NF-κB luciferase reporter genes from Stratagene (La, Jolla, CA, USA) were then transiently transfected into HMC-1 cells using Lipofectamine 2000 reagent from Invitrogen (Carlsbad, CA, USA) according to the manufacturer’s instructions. After 24 h of incubation, the cells were pre-treated with CJ for 1 h and then stimulated with PMA and A23187 for 24 h. Cells were washed in ice-cold PBS and lysed with lysis buffer of Luciferase assay kit from Promega (Madison, WI, USA). Luciferase activity was determined by measuring the total protein and expressed as relative light units per milligram of protein in the cell lysate using a luminometer (Perkin-Elmer).

### Statistical analysis

All data are reported as the mean ± standard deviation (SD). Student’s *t* test was used for single variable comparisons and a *p* value <0.05 was considered to be statistically significant.

## Results

### Effect of CJ and silibinin on cell viability and histamine release in activated mast cells

**Fig. 1:**
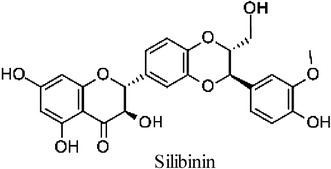
Molecular structure of silibinin

**Fig. 2:**
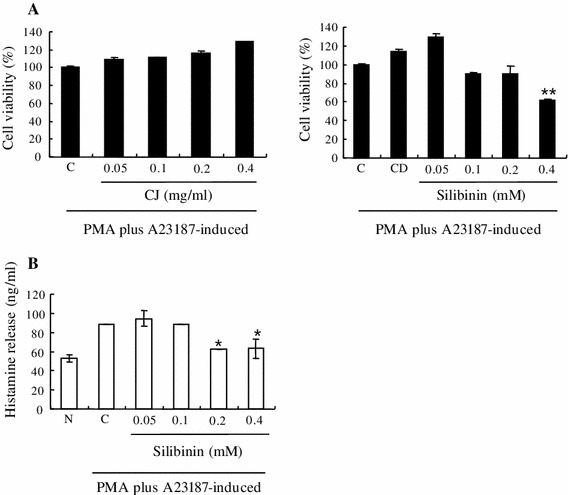
Effect of CJ and silibinin on cell viability and histamine release in activated mast cells. The HMC-1 cells (1 × 10^6^ cells/ml) were pretreated with indicated concentrations of CJ (0.05 to 0.4 mg/ml) or silibinin (0.05–0.4 mM) for 1 h and then incubated with PMA and A23187 for 24 h (**a**) or 6 h (**b**). Cell viability was determined with an MTS assay. Histamine release was measured by enzyme immunoassay. Each data point presents the mean ± SD of three independent experiments (*p < 0.05 and **p < 0.01 compared to control). *N* no treatment, *C* induced control, *CD* induced control with DMSO treatment

Mast cells are activated by PMA and A23187 and secrete inflammatory mediators such as histamine, serotonin, hydrolase, heparin, and prostaglandin. In this study, we investigated the effect of CJ and silibinin (Fig. 1) on cell viability and histamine release in HMC-1 cells. We found that CJ did not affect cell viability at the concentrations used while silibinin decreased cell viability by 30 % at 0.4 mM (Fig. 2a). It should be noted that silibinin increased cell viability by 30 % at 0.05 mM, suggesting that it may have biphasic effect on cell viability. On the other hand, 0.2 mM and 0.4 mM silibinin decreased histamine release induced by PMA plus A23187 (Fig. 2b).

### Effect of CJ and silibinin on PMA plus A23187-stimulated TNF-α expression

**Fig. 3:**
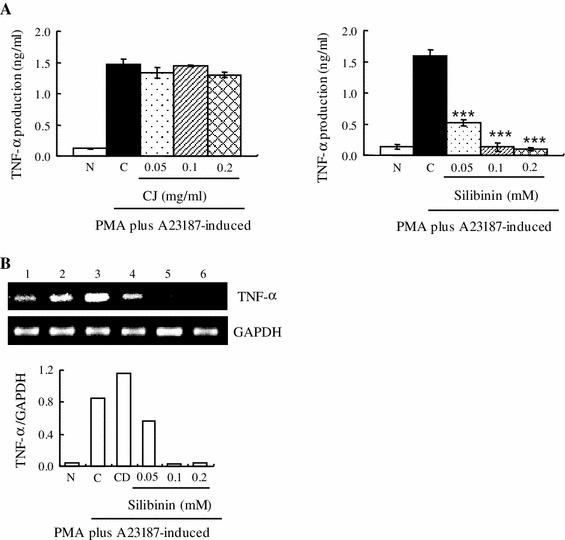
Effect of CJ and silibinin on PMA plus A23187-stimulated TNF-α expression. The HMC-1 cells (1 × 10^6^ cells/ml) were preincubated with various concentrations of CJ (0.05 to 0.2 mg/ml) or silibinin (0.05–0.2 mM) for 1 h and then treated with PMA plus A23187 for 4 h (**a**, **b**). **a** The TNF-α secreted protein level in the supernatant was measured by the ELISA assay. **b** The TNF-α mRNA level was measured by the RT-PCR. *1* Normal cells, *2* control cells, *3* DMSO control cells, *4* silibinin (0.05 mM) + PMA plus A23187, *5* silibinin (0.1 mM) + PMA plus A23187, *6* silibinin (0.2 mM) + PMA plus A23187. Each data point presents the mean ± SD of three independent experiments (***p < 0.001 compared to control). *N* no treatment, *C* induced control, *CD* induced control with DMSO treatment

Pro-inflammatory cytokines are also important factors of allergic inflammations. Therefore, the production and expression of TNF-α were determined by ELISA or RT-PCR to evaluate the effect of CJ and silibinin on the pro-inflammatory cytokines. Due to the cytotoxicity at 0.4 mM of silibinin, the modulation of pro-inflammatory cytokines in treatment of 0.05–0.2 mM of silibinin was observed. We found that CJ did not affect the production level of TNF-α while silibinin significantly decreased TNF-α production induced by PMA plus A23187 (Fig. 3a). Moreover, the mRNA level of TNF-α induced by PMA plus A23187 was also reduced by silibinin (Fig. 3b).

### Effect of CJ and silibinin on PMA plus A23187-stimulated IL-6 expression

**Fig. 4:**
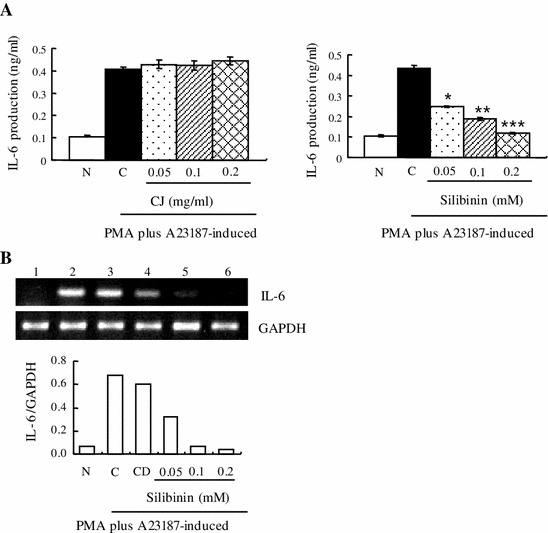
Effect of CJ and silibinin on PMA plus A23187-stimulated IL-6 expression. The HMC-1 cells (1 × 10^6^ cells/ml) were preincubated with various concentrations of CJ (0.05 to 0.2 mg/ml) or silibinin (0.05–0.2 mM) for 1 h and then treated with PMA plus A23187 for 4 h (**a**, **b**). **a** The IL-6 secreted protein level in the supernatant was measured by the ELISA assay. **b** The IL-6 mRNA level was measured by the RT-PCR. *1* Normal cells, *2* control cells, *3* DMSO control cells, *4* silibinin (0.05 mM) + PMA plus A23187, *5* silibinin (0.1 mM) + PMA plus A23187, *6* silibinin (0.2 mM) + PMA plus A23187. Each data point presents the mean ± SD of three independent experiments (*p < 0.05, **p < 0.01, and ***p < 0.001 compared to control). *N* no treatment, *C* induced control, *CD* induced control with DMSO treatment

We determined the production and expression of IL-6 by ELISA or RT-PCR. We found that CJ did not affect the production level of IL-6 while silibinin significantly decreased IL-6 production induced by PMA plus A23187 (Fig. 4a). Moreover, mRNA level of IL-6 induced by PMA plus A23187 was also reduced by silibinin (Fig. 4b).

### Effect of CJ and silibinin on PMA plus A23187-stimulated IL-8 expression

**Fig. 5:**
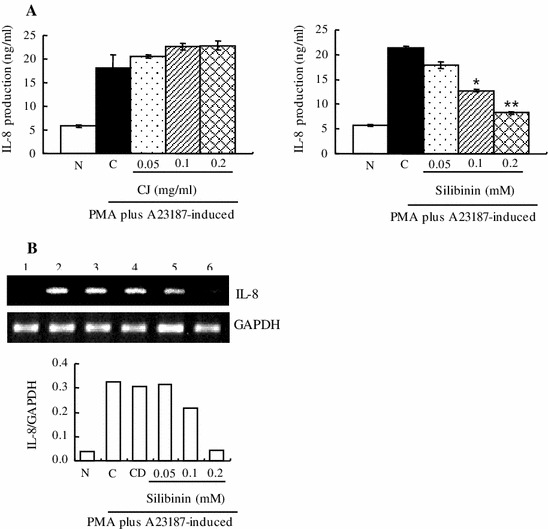
Effect of CJ and silibinin on PMA plus A23187-stimulated IL-8 expression. The HMC-1 cells (1 × 10^6^ cells/ml) were preincubated with various concentrations of CJ (0.05–0.2 mg/ml) or silibinin (0.05–0.2 mM) for 1 h and then treated with PMA plus A23187 for 4 h (**a**, **b**). **a** The IL-8 secreted protein level in the supernatant was measured by the ELISA assay. **b** The IL-8 mRNA level was measured by the RT-PCR. *1* Normal cells, *2* control cells, *3* DMSO control cells, *4* silibinin (0.05 mM) + PMA plus A23187, *5* silibinin (0.1 mM) + PMA plus A23187, *6* silibinin (0.2 mM) + PMA plus A23187. Each data point presents the mean ± SD of three independent experiments (*p < 0.05 and **p < 0.01 compared to control). *N* no treatment, *C* induced control, *CD* induced control with DMSO treatment

We measured the production and expression of IL-8 by ELISA or RT-PCR. We observed that CJ did not change the production level of IL-8 whereas silibinin significantly reduced IL-8 production induced by PMA plus A23187 (Fig. 5a). Furthermore, mRNA level of IL-8 induced by PMA plus A23187 was also decreased by silibinin (Fig. 5b).

### Effect of silibinin on cell viability and LPS-stimulated IL-6 expression in RAW264.7 mouse macrophage cells

**Fig. 6:**
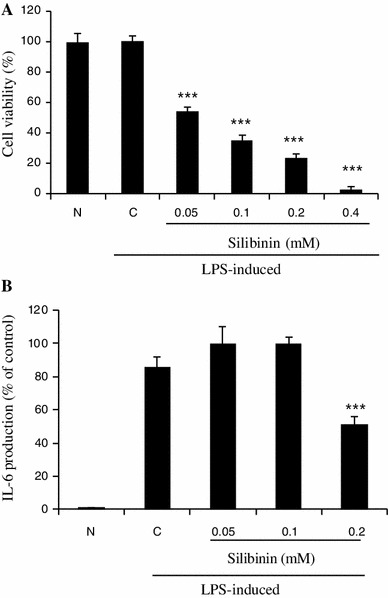
Effect of silibinin on cell viability and LPS-stimulated IL-6 expression in RAW264.7 mouse macrophage cells. The RAW264.7 cells (1 × 10^6^ cells/ml) were pretreated with indicated concentrations of silibinin (0.05 to 0.4 mM) for 1 h and then incubated with LPS for 24 h. **a** Cell viability was determined with an MTS assay. **b** The IL-6 secreted protein level in the supernatant was measured by the ELISA assay. Each data point presents the mean ± SD of three independent experiments (***p < 0.001 compared to control). *N* no treatment, *C* induced control

To verify whether silibinin has a regulatory effect on other cell lines, we performed experiments (MTS and ELISA) in RAW264.7 mouse macrophage cells, which are known to produce pro-inflammatory cytokines as HMC-1 cells do. We found that silibinin suppressed significantly cell growth in a dose-dependent manner (Fig. 6a) and reduced IL-6 production induced by LPS (Fig. 6b).

### Effect of silibinin on PMA plus A23187-stimulated NF-κB activation and IκBα phosphorylation and degradation

**Fig. 7:**
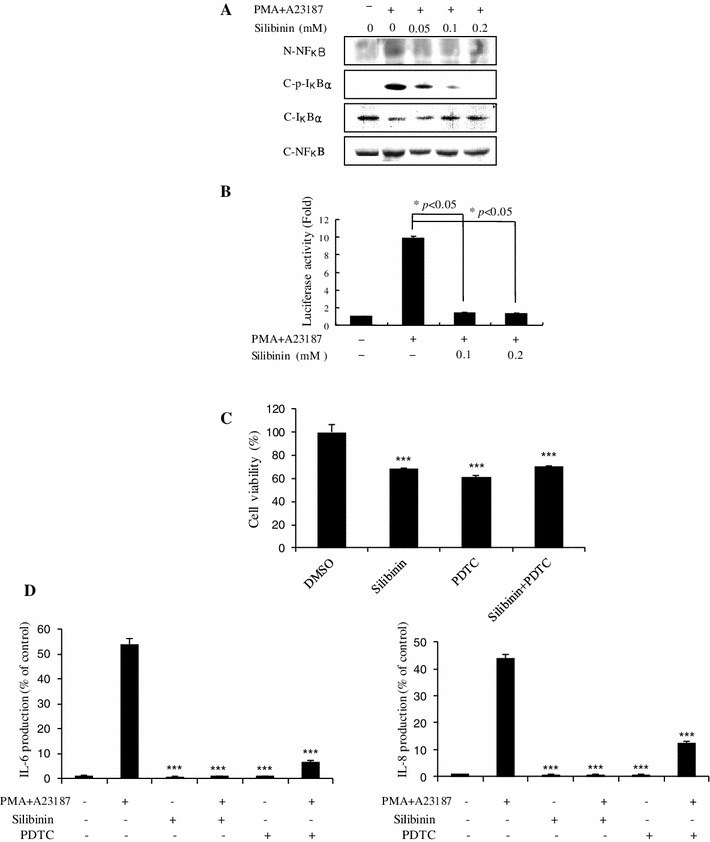
Effect of silibinin on PMA plus A23187-stimulated NF-κB activation and IκBα phosphorylation and degradation. **a** HMC-1 cells (1 × 10^6^ cells/ml) were incubated with silibinin (0.05–0.2 mM) for 1 h and then stimulated with PMA plus A23187 for 2 h. Nuclear and cytoplasmic proteins were isolated by lysis buffer and examined for NF-κB, p-IκBα, and IκBα with Western-blot analysis. *N* nuclear extract, *C* cytosol extract. **b** The HMC-1 cells (1 × 10^6^ cells/ml) were treated with silibinin (0.1–0.2 mM) for 1 h and then stimulated with PMA plus A23187 for 4 h. The NF-κB transcriptional activity was examined with a luciferase assay. *p < 0.05; significantly different from the control value. **c** The HMC-1 cells (1 × 10^6^ cells/ml) were treated with silibinin (0.4 mM), PDTC (40 μM) or silibinin and PDTC for 72 h. Cell viability was determined with an MTS assay. Each data point presents the mean ± SD of three independent experiments (***p < 0.001 compared to control). **d** The IL-6 and IL-8 secreted protein level in the supernatant was measured by the ELISA assay. Each data point presents the mean ± SD of three independent experiments (***p < 0.001 compared to PMA plus A23187)

The expression of pro-inflammatory cytokines such as TNF-α, IL-6, and IL-8 are regulated by NF-κB signaling. Therefore, we examined whether silibinin affects the expression of NF-κB signaling molecules or NF-κB transcriptional activity. We found that silibinin suppressed the expression of nuclear NF-κB induced by PMA plus A23187 (Fig. 7a). We also found that silibinin inhibited the phosphorylation of IκBα in stimulated HMC-1 cells, suggesting that it abrogates the dissociation of IκBα from NF-κB heterodimer (p65 and p50) to suppress NF-κB signaling (Fig. 7a). Consistent with these results, reporter activity of NF-κB was also reduced in response of silibinin pretreatment (*p* < 0.05, Fig. 7b), indicating that silibinin could regulate the expression of pro-inflammatory cytokines such as TNF-α, IL-6, and IL-8 through inactivation of phosphorylation of IκBα and blockade of translocation of NF-κB into the nucleus. To confirm that NF-κB inhibition is related to the effect of silibinin on the functions of mast cells, we performed further experiments using the NF-κB inhibitor pyrrolidine dithiocarbamate (PDTC) in HMC-1 cells. We found that PDTC suppressed HMC-1 cell growth like silibinin (Fig. 7c). PDTC also suppressed IL-6 and IL-8 secretion induced by PMA plus A23187 as seen in Fig. 7d. Hence, silibinin seems to inhibit the functions of mast cells through inhibition of NF-κB signaling pathway.

## Discussion

Silibinin is a flavonolignan found in CJ or milk thistle. Silibinin modulates the insulin-like growth factor (IGF) system by increasing circulating levels of IGF-binding protein 3 and decreasing levels of IGF-I [23]. In preliminary studies, silibinin has been tested in various cancers including lung, colon, oral, and prostate. Silibinin contains antioxidant properties and protects the liver from damage and toxins. However, the activity of silibinin on inflammatory diseases has not been well investigated. Therefore, in the present study, we investigated whether CJ and silibinin have a therapeutic effect on allergic inflammatory disease. Interestingly, silibinin suppressed the growth of induced HMC-1 cells by PMA plus A23187 while CJ did not induce such growth inhibition. In agreement with our data, it was reported that silibinin induces growth inhibition and apoptotic cell death in human lung carcinoma cells [24]. It was also reported that silibinin inhibits cell growth and down-regulates survivin in a laryngeal squamous cell carcinoma cell line [25]. This anti-proliferative activity of silibinin seems to be related to its anti-tumor activity in various cancers. It should be noted that 0.05 mM silibinin induced almost “130 %” cell viability of PMA plus A23187-induced cells (Fig. 2a, right panel). This explains that 0.05 mM silibinin is insufficient to inhibit cell growth promotion induced by PMA plus A23187. Silibinin may also have a biphasic effect for cell growth; it helps growth promotion of induced cells at lower concentration whereas it inhibits cell growth at higher concentration. It is well known that many compounds have a biphasic effect on the cell number. This property of silibinin may require some caution for widespread use. We also observed that silibinin inhibited histamine release in induced HMC-1 cells by PMA plus A23187. In agreement with our data, it was reported that silibinin dose-dependently reduced histamine release from rat peritoneal mast cells (RPMC) activated by compound 48/80 or anti-DNP IgE [26]. This suggests that silibinin displays an anti-allergic effect, inhibiting histamine release. Silibinin also reduced the production and mRNA expression of pro-inflammatory cytokines such as TNF-α, IL-6, and IL-8, while CJ did not induce such a reduction. In agreement with our data, it was reported that silibinin inhibited the secretion of TNF-α and IL-6 in RPMC [26].

Allergic inflammation is classified into the phases of early phase (or type I immediate hypersensitivity) and late-phase reactions, which result in chronic allergic inflammation subsequently. Release of histamine and other mediators following the crosslinking of Fc receptor for IgE (FcεRI) bound IgE to allergen in mast cells is known as early phase reaction. IgE-mediated PCA reaction, as a sensitive reaction to detect small quantities of antibodies, has been used to evaluate the mechanisms of immediate allergy reaction [27]. These early phase responses are followed by late-phase reactions that typically develop 2–9 h after allergen exposure. In late-phase reactions, the recruitment of leucocytes including T-cell and neutrophil is featured [28–30]. The transition to the late-phase reaction is characterized as the recruitment of leukocytes by upregulating mediators such as IL-8 and TNF-α [29, 30]. Mast cell-derived IL-8 is suggested to activate neutrophil in allergic inflammation [31]. In the other study, it was reported that monomeric IgE induced a long-lasting, dose-dependent histamine release, LTC4 production and IL-8 synthesis in mast cells [32]. Also, it was reported that TNF-α plays an essential role in the development of late-phase anaphylactic reaction via a PAF-mediated NF-κB-dependent way and initiates late-phase allergic inflammation [33]. Therefore, our finding of considerable changes in histamine release and significant suppression of pro-inflammatory cytokines suggests that silibinin might have inhibitory effects on allergic inflammation through early phase, transition, and late-phase reaction.

It should be noted that silibinin contains anti-proliferative effect and inhibitory activity on pro-inflammatory cytokines production while CJ did not show such an effect. This result might be due to either the presence of additional molecules in CJ that antagonize the effect of silibinin or insufficient concentration of silibinin in CJ. We determined the content of silibinin in CJ. We found that the content of silibinin in CJ samples was <1 ppb (SRM model) (data not shown). Since the content of silibinin in our CJ sample is small, CJ did not seem to show anti-inflammatory effects. It should also be noted that PMA alone fails to induce degranulation of mast cells. Therefore, we stimulated HMC-1 cells by PMA and A23187 in our experiments. To know whether silibinin has a similar effect on other cell line, we performed further experiments in RAW264.7 mouse macrophage cells, which are known to produce pro-inflammatory cytokines like HMC-1 cells. We found that silibinin suppressed significantly cell growth in a dose-dependent manner and reduced IL-6 production induced by LPS. This suggests that silibinin inhibits the production of pro-inflammatory cytokines in different cell systems.

NF-κB fulfills a central role in the cellular stress response and in inflammation by controlling the expression of a network of genes such as TNF-α, IL-6, and IL-8. Upon infection, microbial pathogens are sensed by the host and activate NF-κB transcription factors via triggering of various sensors, like the Toll-like receptors, which are expressed on cells of the innate immune system, including macrophages, dendritic cells, and mucosal epithelial cells [34, 35]. NF-κB activation is tightly controlled by pathways which can regulate the proteolysis of the inhibitory IκB and IκB-related proteins. In unstimulated cells, NF-κB dimers are sequestered in the cytoplasm via physical association with NF-κB inhibitory proteins, IκBs [34]. Upon stimulation, signal transduction events rapidly lead to the activation of the IκB kinase (IKK) complex, composed of two catalytic subunits (IKKα and IKKβ) and a regulatory subunit, NF-κB essential modulator [34]. Activated IKK phosphorylates IκBα, predominantly via the action of IKKβ, triggering its polyubiquitination and proteasomal degradation and inducing the nuclear translocation of associated NF-κB subunits [34]. NF-κB subunits bind to specific DNA to induce the transcription of target genes. Our results show that silibinin suppressed the nuclear translocation of NF-κB as well as the phosphorylation of IκBα, which might inhibit the expression of the proinflammatory cytokines such as TNF-α, IL-6, and IL-8. It should also be noted that PDTC (an NF-κB inhibitor) suppressed HMC-1 cell growth and reduced IL-6 and IL-8 secretion induced by PMA plus A23187, like silibinin. It is well known that inhibition of NF-κB translocation or Iκ-Bα phosphorylation reduces the expression of pro-inflammatory cytokines such as TNF-α, IL-6, and IL-8. However, there are not many reports regarding the effect of silibinin on the production of pro-inflammatory cytokines in human mast cells. We emphasized in our report that silibinin has an inhibitory effect on the production of pro-inflammatory cytokines through inhibition of NF-κB signaling pathway in HMC-1 human mast cells.

Our data suggest that silibinin inhibits the production of pro-inflammatory cytokines through inhibition of the NF-κB signaling pathway in HMC-1 human mast cells. Our findings indicate that silibinin could be a potential medicine for the treatment of allergic and inflammatory diseases.

## References

[1] Ferrero-Miliani L, Nielsen OH, Andersen PS, Girardin SE (2007). Chronic inflammation: importance of NOD2 and NALP3 in interleukin-1beta generation. Clin Exp Immunol.

[2] Rosenwasser LJ (2011). Current understanding of the pathophysiology of allergic rhinitis. Immunol Allergy Clin North Am..

[3] Rose S, Weld-Moore R, Ghazali N, Newman L (2011). I’ve got hay-fever and my mouth is stinging!. Br Dent J.

[4] Olivera A, Rivera J (2011). An emerging role for the lipid mediator sphingosine-1-phosphate in mast cell effector function and allergic disease. Adv Exp Med Biol.

[5] Kuna P, Reddigari SR, Rucinski D, Oppenheim JJ, Kaplan AP (1992). Monocyte chemotactic and activating factor is a potent histamine-releasing factor for human basophils. J Exp Med.

[6] Wang SL, Malany S, Wang Q, Santos MA, Crowe PD, Maki RA (2006). Histamine induces interleukin-6 expression in the human synovial sarcoma cell line (SW982) through the H1 receptor. Inflamm Res.

[7] Thomas PS (2001). Tumor necrosis factor-alpha: the role of this multifunctional cytokine in asthma. Immunol Cell Biol.

[8] Naka S, Suto H, Kakurai M, Sedgwick JD, Tsai M, Galli SJ (2005). Mast cells enhance T cell activation: importance of mast cell-derived TNF. Proc Natl Acad Sci USA.

[9] Bodreau RT, Hoskin DW, Lin TJ (2004). Phosphatase inhibition potentiates IL-6 production by mast cells in response to FcεRI-mediated activation: involvement of p38 MAPK. J Leukoc Biol.

[10] Murayama T, Mukaida N, Sadanari H, Yamaguchi N, Khabar KS, Tanaka J, Matsushima K, Mori S, Eizuru Y (2000). The immediate early gene 1 product of human cytomegalovirus is sufficient for up-regulation of interleukin-8 gene expression. Biochem Biophys Res Commun.

[11] Kuprsh DV, Udalova IA, Turetskaya RL, Rice NR, Nedospasov SA (1995). Conserved kappa B element located downstream of the tumor necrosis factor alpha gene: distinct NF-kappa B binding pattern and enhancer activity in LPS activated murine macrophages. Oncogene.

[12] Galien R, Evans HF, Garcia T (1996). Involvement of CCAAT/enhancer-binding protein and nuclear factor-kappa B binding site in interleukin-6 promoter inhibition by estrogens. Mol Endocrinol.

[13] Kim DY, Kang SH, Ghil SH (2010). Cirsium japonicum extract induces apoptosis and anti-proliferation in the human breast cancer cell line MCF-7. Mol Med Rep..

[14] Ishida H, Umino T, Tsuji K, Kosuge T (1987). Studies on antihemorrhagic substances in herbs classified as hemostatics in Chinese medicine. VII. On the antihemorrhagic principle in *Cirsium japonicum* DC. Chem Pharm Bull.

[15] Park MK, Rhyu MR, Yoon BK, Kwon H, Jang S, Lee YJ (2008). Modulation of the genomic estrogen receptor pathway by water extracts of *Cirsium japonicum*. Arch Pharm Res..

[16] Kim EY, Jho HK, Kim DI, Rhyu MR (2008). *Cirsium japonicum* elicits endothelium-dependent relaxation via histamine H(1)-receptor in rat thoracic aorta. J Ethnopharmacol.

[17] Yin J, Heo SI, Wang MH (2008). Antioxidant and antidiabetic activities of extracts from *Cirsium japonicum* roots. Nutr Res Pract..

[18] Liu SJ, Luo X, Li DX, Zhang J, Qiu DL, Liu W, She L, Yang ZR (2006). Tumor inhibition and improved immunity in mice treated with flavone from *Cirsium japonicum* DC. Int Immunopharmacol.

[19] Schumann J, Prockl J, Kiemer AK, Vollmar AM, Bang R, Tiegs G (2003). Silibinin protects mice from T cell-dependent liver injury. J Hepatol.

[20] Wu JW, Lin LC, Hung SC, Lin CH, Chi CW, Tsai TH (2008). Hepatobiliary excretion of silibinin in normal and liver cirrhotic rats. Drug Metab Dispos.

[21] Gazák R, Walterová D, Kren V (2007). Silybin and silymarin—new and emerging applications in medicine. Curr Med Chem.

[22] Duan W, Jin X, Li Q, Tashiro S, Onodera S, Ikejima T (2010). Silibinin induced autophagic and apoptotic cell death in HT1080 cells through a reactive oxygen species pathway. J Pharmacol Sci..

[23] Hoh C, Boocock D, Marczylo T, Singh R, Berry DP, Dennison AR, Hemingway D, Miller A, West K, Euden S, Garcea G, Farmer PB, Steward WP, Gescher AJ (2006). Pilot study of oral silibinin, a putative chemopreventive agent, in colorectal cancer patients: silibinin levels in plasma, colorectum, and liver and their pharmacodynamic consequences. Clin Cancer Res.

[24] Sharma G, Singh RP, Chan DC, Agarwal R (2003). Silibinin induces growth inhibition and apoptotic cell death in human lung carcinoma cells. Anticancer Res.

[25] Bang CI, Paik SY, Sun DI, Joo YH, Kim MS (2008). Cell growth inhibition and down-regulation of survivin by silibinin in a laryngeal squamous cell carcinoma cell line. Ann Otol Rhinol Laryngol..

[26] Choi YH, Yan GH (2009). Silibinin attenuates mast cell-mediated anaphylaxis-like reactions. Biol Pharm Bull.

[27] Harada M, Nagata M, Takeuchi M (1988). Production of passive cutaneous anaphylaxis (PCA) and reversed PCA by rat IgE antibody in the mouse. Experientia.

[28] Valenta R (2002). The future of antigen-specific immunotherapy of allergy. Nat Rev Immunol.

[29] Galli SJ, Tsai M, Piliponsky AM (2008). The development of allergic inflammation. Nature.

[30] Bischoff SC (2007). Role of mast cells in allergic and non-allergic immune responses: comparison of human and murine data. Nat Rev Immunol.

[31] Lippert U, Moller A, Welker P, Artuc M, Henz BM (2000). Inhibition of cytokine secretion from human leukemic mast cells and basophils by H1- and H2-receptor antagonists. Exp Dermatol.

[32] Cruse G, Kaur D, Yang W, Duffy SM, Brightling CE, Bradding P (2005). Activation of human lung mast cells by monomeric immunoglobulin E. Eur Respir J.

[33] Choi IW, Kim YS, Kim DK, Choi JH, Seo KH, Im SY, Kwon KS, Lee MS, Ha TY, Lee HK (2003). Platelet-activating factor-mediated NF-kappaB dependency of a late anaphylactic reaction. J Exp Med.

[34] Mohamed MR, McFadden G (2009). NFκB inhibitors: strategies from poxviruses. Cell Cycle.

[35] Medzhitov R (2001). Toll-like receptors and innate immunity. Nat Rev Immunol.

